# Lactic Acid Fermentation of Carrot Juice: Effects of LAB Strains, Total Soluble Solids, Inoculum Level, and Fermentation Time

**DOI:** 10.1002/fsn3.71917

**Published:** 2026-05-18

**Authors:** Thanh Viet Nguyen, Chi Khang Van, Ho Thi Uyen Em, Luu Minh Chau, Nguyen Ngoc Thanh, Huynh Xuan Phong

**Affiliations:** ^1^ Institute of Applied Technology and Sustainable Development Nguyen Tat Thanh University Ho Chi Minh City Vietnam; ^2^ High Technology Development Center Nguyen Tat Thanh University Ho Chi Minh City Vietnam; ^3^ Institute of Food and Biotechnology Can Tho University Can Tho City Vietnam

**Keywords:** carrot juice, fermented beverages, inoculum level, LAB, lactic acid fermentation

## Abstract

Carrot juice is a promising substrate for developing functional fermented beverages; however, optimal conditions for lactic acid fermentation remain insufficiently defined. This study investigated the effects of lactic acid bacteria (LAB) strains, juice dilution ratio, initial total soluble solids (TSS, °Brix), inoculum level, and fermentation time on the fermentation performance and quality of carrot juice. Ten LAB strains previously isolated from fermented tomato products were screened, among which strain TO35 exhibited the highest lactic acid production (12.37 ± 0.45 g/L). Juice dilution significantly influenced fermentation efficiency, with undiluted and 1:1 diluted formulations showing superior acidification performance compared with higher dilution levels. The combination of 15°Brix and 3% (v/v) inoculum resulted in maximal lactic acid production (14.10 g/L) and high LAB viability. Fermentation time markedly affected biochemical and sensory attributes. Although prolonged fermentation (48–72 h) further increased lactic acid production, it led to excessive acidification and reduced sensory acceptability. Therefore, fermentation for 24 h yielded the highest sensory acceptance, characterized by typical carrot color, balanced aroma, and minimal visible sedimentation, contributing to acceptable sensory quality. Overall, lactic acid fermentation of carrot juice was optimally achieved using a 3% TO35 inoculum at 15°Brix with a fermentation duration of 24 h. These findings provide useful insights for the development of carrot‐based lactic fermented beverages with improved sensory quality and functional potential.

## Graphical abstract

Graphical overview of lactic acid fermentation of carrot juice using LAB strain TO35 under optimized conditions (1:1 dilution ratio, 15°Brix, 3% inoculum, and 24 h fermentation), resulting in enhanced lactic acid production, LAB viability, antioxidant activity, and sensory quality.

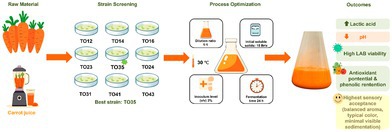

## 1 Introduction

Carrots ( *Daucus carota* L.) are among the most widely consumed vegetables worldwide and are valued for their high contents of carotenoids, vitamins, minerals, dietary fiber, and phenolic compounds. These bioactive constituents are associated with various health benefits, including antioxidant protection, reduced cardiovascular disease risk, improved visual function, and enhanced immune responses (Nagraj et al. 2020; Arshad et al. 2025). However, fresh carrots and carrot juice are highly susceptible to enzymatic degradation, oxidative reactions, and nutrient losses during processing and storage, which significantly limit shelf life and functional quality (Hwang et al. 2023). Consequently, there is increasing interest in processing strategies that not only preserve but also enhance the nutritional and sensory attributes of carrot‐based products.

Lactic acid fermentation mediated by lactic acid bacteria (LAB) is widely recognized as an effective approach to improve nutritional quality, microbiological safety, and sensory characteristics of plant‐based foods and beverages. During fermentation, LAB produce organic acids, aroma‐active compounds, exopolysaccharides, and various bioactive metabolites that contribute to flavor development, texture modification, and functional enhancement (Nguyen et al. 2025; Sivamaruthi et al. 2025). Recent studies on fermented vegetable juices, including beetroot, cabbage, tomato, and pumpkin, have demonstrated enhanced antioxidant capacity, improved stability of sensitive phytochemicals, and increased consumer acceptability (Žvirdauskienė et al. 2025). In parallel, global consumer demand for plant‐based fermented beverages has increased steadily due to their dairy‐free nature, perceived health benefits, and potential probiotic effects. Despite growing scientific and commercial interest, systematic investigations into the lactic acid fermentation of carrot juice remain limited. In particular, the interactive effects of LAB strain selection and key processing parameters such as juice dilution ratio, initial soluble solids (°Brix), inoculum concentration, and fermentation time have not been comprehensively evaluated. Although carrot juice contains abundant fermentable sugars, carotenoids, and phenolic acids that support LAB growth and metabolite production (Wuyts et al. 2018; Raichurkar et al. 2026), fermentation performance is highly strain‐dependent and strongly influenced by processing conditions, which collectively determine acidification kinetics, microbial dynamics, and sensory quality (Mengesha et al. 2022; Huynh et al. 2023; Abbaspour 2024; Xie et al. 2026). Most previous studies have focused on single‐factor assessments, highlighting a clear need for multi‐factor, strain‐specific optimization approaches that integrate both physicochemical and sensory attributes.

Therefore, this study aimed to systematically evaluate lactic acid fermentation of carrot juice using 10 LAB strains previously isolated from fermented tomato products. Among these, strain TO35 exhibited the highest lactic acid production (12.37 ± 0.45 g/L). A stepwise optimization approach was applied to investigate the combined effects of juice dilution ratio, initial °Brix level, inoculum concentration, and fermentation time. The objective was to identify optimal fermentation conditions that maximize lactic acid production and microbial viability while enhancing antioxidant potential and sensory acceptability. The findings provide a scientifically robust basis for developing high‐quality functional fermented carrot beverages aligned with current nutritional trends and consumer preferences.

## 2 Materials and Methods

### 2.1 Materials

Fresh orange carrots ( *Daucus carota* L.), exhibiting typical orange pigmentation due to β‐carotene, were purchased from local markets in Ninh Kieu, Can Tho City, Vietnam, and processed on the day of collection. Ten LAB strains (TO12, TO14, TO16, TO23, TO24, TO31, TO35, TO41, TO43, and TO52), previously isolated from fermented tomato products (Le et al. 2023), were obtained from the Culture Collection of the Laboratory of Industrial Microbiology, Institute of Food and Biotechnology, Can Tho University (Can Tho City, Vietnam). The strains were originally isolated using serial dilution and cultivation on De Man–Rogosa–Sharpe (MRS) agar at 37°C. Preliminary identification was based on colony morphology and cell characteristics, followed by confirmation using Gram staining, catalase test, oxidase test, and motility assessment as described in Le et al. (2023). For preservation, the strains were stored in MRS broth supplemented with 20% (v/v) glycerol at −80°C for long‐term storage, while working cultures were maintained on MRS agar at 4°C and periodically subcultured for experimental use.

Analytical‐grade chemicals, including sodium hydroxide (NaOH; Cemaco, Vietnam), sodium chloride (NaCl; Xilong Scientific, China), hydrochloric acid (HCl; Xilong Scientific, China), gallic acid (Sigma‐Aldrich, Germany), and Folin–Ciocalteu reagent (Sigma‐Aldrich, Germany), were used for analytical determinations. De Man‐Rogosa‐Sharpe (MRS) medium (HiMedia, India) was employed for LAB activation, cultivation, and viability assessment.

### 2.2 Preparation of Carrot Juice

Fresh carrots were washed thoroughly, peeled, trimmed, and juiced using a mechanical extractor. The juice was filtered through a double‐layer muslin cloth to remove coarse pulp, and the initial pH was adjusted to 6.5 using sterile NaOH solution. To reduce native microbial load and limit enzymatic browning, sodium metabisulfite (NaHSO_3_) was added at a concentration of 0.1% (w/v) and maintained for 2 h at ambient temperature (25°C ± 2°C), following sulfite‐based pretreatment methods commonly applied in vegetable juice processing (Ren et al. 2022). Preliminary experiments confirmed that the application of sodium metabisulfite at 0.1% (w/v) did not adversely affect LAB viability or lactic acid production during subsequent fermentation. After pretreatment, the juice was aseptically transferred into sterile glass bottles and used immediately for subsequent experiments. The glass bottles were washed, dried, and sterilized by autoclaving at 121°C for 15 min prior to use. The transfer was performed under aseptic conditions using sterile utensils in a laminar airflow cabinet to minimize microbial contamination. The bottles were immediately sealed after filling. Total soluble solids (TSS, °Brix) were measured using a digital refractometer (Atago, Japan), and sterile sucrose solution was added when necessary to obtain the desired Brix levels.

### 2.3 LAB Strains and Inoculum Preparation

Ten LAB strains previously isolated from fermented tomato products and maintained in the Culture Collection of the Laboratory of Industrial Microbiology, Institute of Food and Biotechnology, were used in this study. The strains included TO12, TO14, TO16, TO23, TO24, TO31, TO35, TO41, TO43, and TO52. Each strain was activated by inoculating a loopful of culture into 10 mL of de Man‐Rogosa‐Sharpe (MRS) broth (HiMedia, India) and incubating at 30°C for 24 h. Activated cultures were subsequently propagated in fresh MRS broth to obtain high‐density starter cultures. Cell density was standardized to approximately 10^9^ CFU/mL by determining viable cell counts using serial dilution and plate counting on MRS agar. Based on the measured counts, the cultures were adjusted by dilution with sterile MRS broth or further incubation to achieve the target cell density prior to use as inoculum. The prepared cultures were used as inocula at 1% (v/v) for all screening and optimization experiments.

### 2.4 Screening of LAB Strains

Screening was performed to identify the most suitable LAB strain for carrot juice fermentation. Ten LAB strains (TO12, TO14, TO16, TO23, TO24, TO31, TO35, TO41, TO43, and TO52), previously isolated from fermented tomato products, were evaluated individually. Fresh carrot juice was prepared as described in Section 2.2, adjusted to pH 6.5, and treated with sodium metabisulfite (NaHSO_3_, 0.1% w/v) for 2 h to reduce native microbial load. After treatment, 100 mL of juice was aseptically dispensed into sterile glass bottles. Each LAB strain was inoculated at 1% (v/v) using standardized starter cultures prepared as described in Section 2.3. Fermentation was conducted at 30°C for 48 h under static, non‐aerated conditions in sealed glass bottles, corresponding to facultative anaerobic conditions typical for LAB fermentation. At the end of fermentation, samples were analyzed for TSS (°Brix), pH, and lactic acid concentration according to the analytical procedures described in Section 2.8. The strain exhibiting the highest lactic acid production and the greatest pH reduction was selected as the optimal starter culture for subsequent optimization experiments (Sections 2.5, 2.7).

### 2.5 Effect of Juice Dilution

The effect of juice dilution on the fermentation performance of carrot juice was evaluated using four dilution ratios: 1:0, 1:1, 1:2, and 1:3 (juice: water, v/v). Fresh carrot juice prepared according to Section 2.2 had an initial TSS of 8.03° ± 0.15°Brix, as measured using a digital refractometer (Atago, Japan). The juice was then diluted with sterile distilled water to the designated ratios. The soluble solids content of each mixture was adjusted to 15°Brix using sterile sucrose solution, and the pH was adjusted to 6.5. To ensure uniform pretreatment across samples, sodium metabisulfite (NaHSO_3_ 0.1% w/v) was added and maintained for 2 h as described previously (Section 2.2). After pretreatment, 100 mL of each diluted juice was aseptically transferred into sterile glass bottles. The LAB strain selected from the screening step (Section 2.4) was inoculated at 1% (v/v), corresponding to approximately 10^9^ CFU/mL. Fermentation was conducted at 30°C for 48 h under static conditions. At the end of fermentation, TSS (°Brix), pH, and lactic acid concentration were determined following the analytical procedures described in Section 2.8. Sensory evaluation (color, aroma, and overall acceptability) was conducted as a complementary assessment to support the selection of an appropriate dilution ratio for subsequent optimization experiments.

### 2.6 Effect of TSS and Inoculum Levels

The combined effects of initial soluble solids and inoculum concentration on lactic acid fermentation of carrot juice were investigated using a two‐factor experimental design. Six TSS levels (9°, 12°, 15°, 18°, 21°, and 24°Brix) were prepared by adjusting the soluble solids of carrot juice with sterile sucrose solution, followed by pH adjustment to 6.5. Three inoculum levels (1%, 2%, and 3%, v/v) of the selected LAB strain TO35 were evaluated. For each treatment combination (3 inoculum levels × 6 TSS levels = 18 treatments), 100 mL of carrot juice was dispensed into sterile fermentation bottles. The juice was treated with sodium metabisulfite (NaHSO_3_, 0.1% w/v) for 2 h to ensure uniform pretreatment across samples. The inoculum used for each treatment was prepared from an actively growing culture standardized to approximately 10^9^ CFU/mL. Fermentation was carried out at 30°C under static, non‐agitated, non‐aerated conditions in sealed glass bottles. Fermentation was monitored for up to 72 h, and samples were collected at defined time intervals (0, 24, 48, and 72 h) to evaluate fermentation kinetics. Unless otherwise stated, a fermentation duration of 48 h was applied for comparative analyses, while 24 h was identified as the optimal condition in terms of sensory quality within the acceptable fermentation range. Samples were collected before and after fermentation to determine TSS (°Brix), pH, lactic acid concentration, total phenolic content, and viable cell counts following the analytical procedures described in Section 2.8. Sensory evaluation was also conducted to assess color, aroma, flavor, and overall acceptability across treatments. The optimal combination of Brix level and inoculum concentration was determined based on lactic acid production, microbial growth, biochemical attributes, and sensory performance.

### 2.7 Effect of Fermentation Time

The effect of fermentation time on the biochemical, microbial, and sensory characteristics of fermented carrot juice was evaluated at six time points: 12, 24, 36, 48, 60, and 72 h. Carrot juice was prepared using the optimal dilution ratio and initial °Brix level determined in previous experiments (Sections 2.5 and 2.6), adjusted to pH 6.5, and pretreated with sodium metabisulfite (NaHSO_3_, 0.1% w/v) for 2 h as described previously (Section 2.2). A volume of 100 mL of the treated juice was aseptically transferred into sterile glass bottles. The selected LAB strain (TO35) was standardized to approximately 10^9^ CFU/mL and inoculated at 3% (v/v), corresponding to the optimal inoculum level determined in Section 2.6. Fermentation was conducted at 30°C under the conditions described in Section 2.6. Samples were collected at each fermentation time point (12, 24, 36, 48, 60, and 72 h) for subsequent analyses. All physicochemical, microbiological, and sensory evaluations were performed as described in Section 2.8. Viable LAB populations were determined and expressed as log CFU/mL using MRS agar. Sensory evaluation (color, aroma, flavor, and overall acceptability) was conducted as a complementary assessment to support the selection of an appropriate fermentation duration that provided optimal product quality.

### 2.8 Analytical Measurements

#### 2.8.1 Determination of Total Soluble Solids (°Brix)

Total soluble solids (°Brix) were determined using a digital refractometer (Atago PAL‐1, Japan) according to AOAC Official Method (AOAC International 2023a). Juice samples were filtered through a fine mesh to remove coarse particles prior to measurement. All measurements were performed at room temperature (25°C ± 2°C), and results were expressed as the mean of three independent replicates.

#### 2.8.2 Measurement of pH

The pH of carrot juice before and after fermentation was measured using a calibrated digital pH meter (Model HI2211; Hanna Instruments, Woonsocket, RI, USA). Calibration was performed prior to each measurement using standard buffer solutions at pH 4.0 and 7.0, following the (AOAC International 2023b).

#### 2.8.3 Determination of Lactic Acid Concentration

Lactic acid concentration was quantified using the standard acid–base titration method as described by AOAC Official Method 947.05 (AOAC International 2023c). Briefly, a 10 mL aliquot of fermented juice was diluted with 40 mL of distilled water and titrated with 0.1 N NaOH using phenolphthalein as an indicator until a persistent pale pink color appeared. Lactic acid content was calculated based on NaOH consumption using the equivalent weight of lactic acid (90.08 g/mol), assuming a 1:1 stoichiometric neutralization reaction. The lactic acid concentration was expressed as grams per liter (g/L).

#### 2.8.4 Total Phenolic Content (TPC)

Total phenolic content was determined using the Folin–Ciocalteu colorimetric method, originally described by Singleton and Rossi (1965) and widely validated and applied in recent studies (Phong et al. 2025). The method was applied with minor modifications. Briefly, 0.5 mL of juice sample was mixed with 2.5 mL of Folin–Ciocalteu reagent diluted 1:10 (v/v) with distilled water and allowed to react for 5 min. Subsequently, 2.0 mL of 7.5% (w/v) sodium carbonate solution was added, and the mixture was incubated in the dark for 30 min at room temperature. Absorbance was measured at 765 nm using a UV–Vis spectrophotometer (Cary 60, Agilent Technologies, USA). Results were expressed as milligrams of gallic acid equivalents per liter (mg GAE/L) based on a gallic acid calibration curve.

#### 2.8.5 Antioxidant Activity

Antioxidant activity was determined using the DPPH method according to Brand‐Williams et al. (1995). A 1 mL aliquot of the diluted sample was mixed with 3 mL of 0.1 mM DPPH solution in methanol and incubated in the dark for 30 min. Absorbance was measured at 517 nm against a methanol blank. Radical scavenging activity (RSA, %) was calculated as follows:

$$\mathrm{RSA}\;\left(\%\right)=\left(\frac{A_{0}-A_{1}}{A_{0}}\;\right)\times 100$$

where *A*_0_ is the absorbance of the control and *A*_1_ is the absorbance of the sample.

#### 2.8.6 Viable Cell Counts

Viable LAB were enumerated using the standard plate count method on MRS agar following ISO 15214:1998 (International Organization for Standardization). Samples were serially diluted with sterile 0.85% physiological saline, spread‐plated in duplicate, and incubated at 30°C for 48 h. Results were expressed as log CFU/mL.

### 2.9 Sensory Evaluation

Sensory evaluation was conducted to assess the effects of fermentation conditions on the organoleptic quality of fermented carrot juice. The procedure followed the Vietnamese standard TCVN 3215–79 for sensory quality assessment and general hedonic testing principles described by Phong et al. (2025). A panel of nine trained assessors (aged 22–35 years; four males and five females), experienced in evaluating fermented vegetable beverages, participated in the evaluation. The use of a trained panel of this size is consistent with established sensory evaluation practices, where smaller panels are acceptable when assessors are well‐trained and familiar with the product category. Samples (30 mL) were served at room temperature under uniform lighting in individual booths. Four sensory attributes (color, aroma, flavor, and overall acceptability) were evaluated using a 5‐point hedonic scale, where 1 = “dislike very much,” 2 = “dislike,” 3 = “neither like nor dislike,” 4 = “like,” and 5 = “like very much.” Each sample was coded with a random three‐digit number and presented in a randomized order to minimize positional and expectation bias. Panelists were instructed to rinse their mouths with drinking water between samples. Mean sensory scores for each attribute were used for statistical comparison among treatments and integrated with physicochemical parameters to support the selection of optimal fermentation conditions.

### 2.10 Statistical Analysis

All experiments were conducted in triplicate, and results were expressed as mean ± standard deviation. Data were analyzed according to a completely randomized design appropriate for each experimental setup. One‐way analysis of variance (ANOVA) was applied to determine significant differences among treatments. When ANOVA indicated significant effects (*p* < 0.05), mean comparisons were performed using the Least Significant Difference (LSD) test. Statistical analyses were carried out using Minitab Statistical Software (version 19; Minitab LLC, USA). Graphs were produced using Microsoft Excel.

## 3 Results and Discussion

### 3.1 Screening of LAB Strains

Ten LAB strains isolated (TO12, TO14, TO16, TO23, TO24, TO31, TO35, TO41, TO43, and TO52) were evaluated to identify the most efficient starter culture for lactic acid fermentation of carrot juice. Substantial strain‐dependent variations were observed in lactic acid production, pH reduction, and changes in soluble solids after 48 h of fermentation (Table 1). Such heterogeneity is common in fruit‐ and vegetable‐based fermentations, where LAB performance is strongly influenced by differences in metabolic pathways, carbohydrate utilization efficiency, and tolerance to plant‐derived phytochemicals (Dissanayake et al. 2025; Gangakhedkar et al. 2025).

**TABLE 1:** Lactic acid concentration, pH, and soluble solids (°Brix) of carrot juice fermented with different LAB strains after 48 h of fermentation.

LAB strain	Lactic acid (g/L)	pH	Soluble solids (°Brix)
TO12	10.43^de^ ± 0.34	3.70^de^ ± 0.02	13.17^b^ ± 0.29
TO14	12.15^ab^ ± 0.23	3.68^e^ ± 0.02	13.00^b^ ± 0.06
TO16	10.43^de^ ± 0.26	3.83^ab^ ± 0.02	13.00^b^ ± 0.05
TO23	10.95^cd^ ± 0.26	3.74^de^ ± 0.03	13.00^b^ ± 0.07
TO24	9.00^f^ ± 0.00	3.75^cd^ ± 0.04	14.00^a^ ± 0.04
TO31	9.68^ef^ ± 0.45	3.87^ab^ ± 0.03	13.33^b^ ± 0.29
TO35	12.38^a^ ± 0.45	3.60^f^ ± 0.03	12.83^b^ ± 0.29
TO41	10.35^de^ ± 0.23	3.81^bc^ ± 0.03	13.00^b^ ± 0.06
TO43	9.83^ef^ ± 0.34	3.89^a^ ± 0.02	13.17^b^ ± 0.29
TO52	11.33^bc^ ± 0.13	3.72^de^ ± 0.01	13.00^b^ ± 0.05

*Note:* Values are expressed as mean ± standard deviation (*n* = 3). Different superscript letters within the same column indicate statistically significant differences at the 95% confidence level (*p* < 0.05) according to LSD test.

Lactic acid concentrations ranged from 9.00 to 12.38 g/L, indicating that all strains were capable of fermenting carrot juice, although with distinct efficiencies. Strain TO35 produced the highest lactic acid level (12.38 g/L), suggesting a highly active homofermentative pathway and efficient conversion of hexose sugars into lactic acid. Strains TO14 (12.15 g/L) and TO52 (11.33 g/L) also showed strong acidification capacity, whereas TO24 and TO43 produced the lowest levels (9.00–9.83 g/L). This pattern is consistent with earlier studies reporting pronounced strain‐specific variation during the fermentation of carrot, beetroot, and tomato juices (Xu et al. 2020; Foss et al. 2023; Tuerhong et al. 2024), highlighting the critical role of metabolic flexibility in vegetable substrates. All strains effectively reduced the initial pH (6.5) to final values ranging from 3.60 to 3.89. The lowest final pH was observed for TO35 (3.60), aligning with its highest lactic acid accumulation. A final pH below 4.0 is considered essential for inhibiting spoilage microorganisms and ensuring microbial stability in fermented beverages (Mafe et al. 2024). Strains that produced lower amounts of lactic acid (e.g., TO24, TO43) exhibited correspondingly higher final pH values (3.83–3.89), indicating weaker acidification kinetics. This inverse correlation between lactic acid formation and final pH is well established in LAB fermentation systems, where rapid lactate accumulation drives extracellular acidification (Zhang et al. 2022). All strains reduced the TSS of carrot juice from 15°Brix to 13.0°–13.3°Brix, confirming active utilization of fermentable sugars for energy production and biomass formation. Although the extent of reduction was moderate, the trend aligned with typical sugar consumption patterns observed in LAB‐mediated vegetable fermentations (Xu et al. 2020). Strains with higher acidification performance (TO35, TO14, TO52) tended to exhibit slightly greater decreases in TSS, suggesting more efficient carbohydrate metabolism. The distinct fermentation behaviors observed among strains can be explained by several underlying mechanisms: LAB strains differ in their phosphotransferase systems (PTS), which regulate the internalization of glucose, fructose, and sucrose. This enhanced performance may be associated with more efficient sugar transport systems and metabolic activity in strain TO35, enabling faster glycolytic flux and enhanced lactic acid production (Somavanshi et al. 2016; Wang et al. 2019). Carrot juice contains phenolic acids, terpenoids, and carotenoids that may impose oxidative or membrane stress on microbial cells. LAB adapted to plant‐based environments often express stronger stress‐response mechanisms, including enhanced antioxidative and membrane‐protective systems (Zhang et al. 2019). Such traits may contribute to the superior performance of TO35 and TO14. Differences in acid tolerance also influence fermentation outcomes. Some strains maintain intracellular pH homeostasis and membrane integrity more effectively as external pH declines, allowing sustained metabolic activity and prolonged lactic acid synthesis (Rault et al. 2009). This mechanism likely explains the more robust acidification exhibited by TO35 compared with TO24 and TO43. The superior fermentation performance of TO35 is consistent with characteristics reported for LAB strains commonly associated with plant‐based fermentations, which are widely recognized for their high sugar utilization efficiency and phenolic tolerance in vegetable substrates (Wang et al. 2019; Zhang et al. 2019). The range of lactic acid concentrations observed in this study (9.00–12.38 g/L) is comparable to values reported for fermented carrot and beetroot juices (Foss et al. 2023), confirming both methodological consistency and the suitability of carrot juice as a fermentation substrate. Based on lactic acid production, pH reduction, and °Brix decrease, strain TO35 demonstrated the most desirable fermentation characteristics. It was therefore selected as the optimal starter culture for subsequent optimization experiments, including assessments of dilution ratio (Section 3.2), Brix level and inoculum concentration (Section 3.3), and fermentation time (Section 3.4). Sensory evaluation was intentionally excluded at this screening stage, as the primary objective was to identify the strain with the strongest biochemical fermentation performance. Although no universally harmonized standard exists specifically for lactic acid–fermented vegetable juices, product quality is commonly evaluated based on parameters such as pH (typically < 4.0), titratable acidity, microbial safety, and sensory acceptability. In addition, a viable LAB population of at least 10^6^–10^7^ CFU/mL is generally recommended to ensure product stability and potential functional benefits (Boyte et al. 2023; Wei et al. 2024). In the present study, the observed lactic acid production and viable cell counts were within these commonly accepted ranges, confirming the suitability of carrot juice as a substrate for LAB fermentation. In this study, viable LAB counts reached approximately 9–10 log CFU/mL after fermentation, exceeding the minimum recommended level for fermented vegetable beverages.

### 3.2 Effect of Juice Dilution Ratio on Fermentation Performance

The dilution ratio of carrot juice significantly influenced lactic acid fermentation performance, as reflected by changes in lactic acid concentration, pH, and TSS after 48 h of fermentation (Table 2). Increasing dilution levels resulted in a marked decline in lactic acid production, accompanied by a progressive increase in final pH values, indicating reduced acidification efficiency under diluted conditions.

**TABLE 2:** Fermentation characteristics of carrot juice at different dilution ratios after 48 h.

Dilution ratio (v/v)	Lactic acid (g/L)	pH	Soluble solids (°Brix)
1:0	11.62^a^ ± 0.34	3.42^c^ ± 0.04	11.00^c^ ± 0.05
1:1	11.33^a^ ± 0.34	3.48^c^ ± 0.02	11.17^c^ ± 0.23
1:2	7.20^b^ ± 0.10	4.17^b^ ± 0.03	14.00^b^ ± 0.05
1:3	4.43^c^ ± 0.52	4.65^a^ ± 0.03	14.83^a^ ± 0.23

*Note:* Values represent mean ± standard deviation (*n* = 3). Different superscript letters within a column indicate significant differences at *p* < 0.05 (one‐way ANOVA followed by LSD test).

At dilution ratios of 1:0 and 1:1 (juice: water, v/v), lactic acid concentrations remained relatively high (11.62 and 11.33 g/L, respectively), suggesting sufficient availability of fermentable sugars and nutrients to support LAB metabolism. In contrast, further dilution to ratios of 1:2 and 1:3 led to a substantial reduction in lactic acid production (7.20 and 4.43 g/L, respectively), likely due to nutrient limitation and diminished metabolic activity of LAB in more diluted substrates. Consistent with these observations, final pH values increased with increasing dilution ratio. Samples fermented at a 1:3 dilution exhibited the highest final pH (4.65), which may compromise microbial stability and product shelf life. Similar dilution‐dependent effects on LAB fermentation efficiency have been reported in other vegetable juices, including carrot, beetroot, and cabbage, where excessive dilution negatively affected carbohydrate availability and buffering capacity, thereby limiting acidification (Swain et al. 2014; Xu et al. 2020; Janiszewska‐Turak et al. 2023). TSS showed an inverse trend, increasing with higher dilution ratios due to reduced sugar consumption relative to initial concentrations. Although TSS reduction was modest at lower dilution ratios, the observed pattern was consistent with typical LAB‐mediated vegetable juice fermentations, where carbohydrate utilization directly governs acid production and fermentation intensity (Qiao et al. 2022). Considering lactic acid production, pH reduction, and overall fermentation efficiency, the 1:1 dilution ratio provided a balanced fermentation matrix and was therefore selected as the optimal condition for subsequent optimization experiments involving TSS level and inoculum concentration.

### 3.3 Combined Effects of Initial TSS Level and Inoculum Concentration

The combined effects of initial TSS and inoculum concentration on the fermentation performance of carrot juice are presented in Table 3. Both factors exerted significant influences on lactic acid production, pH reduction, microbial growth, and total phenolic content, with clear interaction effects observed between substrate concentration and inoculum level.

**TABLE 3:** Fermentation characteristics of carrot juice at different initial Brix levels and inoculum concentrations after 48 h.

Initial Brix (°Brix)	Inoculum level (% v/v)	Lactic acid (g/L)	Soluble solids (°Brix)	pH	Total phenolic content (mg GAE/L)	Viable cell count (log CFU/mL)
9	1	10.88^de^ ± 0.27	7.93^d^ ± 0.09	3.53^c^ ± 0.04	6.11^b^ ± 0.26	9.48^bc^ ± 0.06
	2	10.35^fg^ ± 0.31	6.83^f^ ± 0.09	3.49^cd^ ± 0.03	5.36^d^ ± 0.01	9.64^ab^ ± 0.05
	3	11.48 ^cd^ ± 0.18	6.17^g^ ± 0.23	3.39^de^ ± 0.02	5.78^c^ ± 0.13	9.73^a^ ± 0.04
12	1	11.33^cd^ ± 0.11	10.01^b^ ± 0.23	3.39^de^ ± 0.05	5.62^c^ ± 0.02	9.72^a^ ± 0.05
	2	11.03^de^ ± 0.36	9.00^c^ ± 0.08	3.37^e^ ± 0.01	5.20^d^ ± 0.01	9.69^ab^ ± 0.04
	3	11.63^c^ ± 0.28	9.00^c^ ± 0.07	3.37^e^ ± 0.02	5.50^cd^ ± 0.01	9.75^b^ ± 0.05
15	1	12.07^b^ ± 0.11	11.16^a^ ± 0.23	3.33^e^ ± 0.01	5.46^cd^ ± 0.03	9.76^a^ ± 0.06
	2	12.75^ab^ ± 0.27	11.17^a^ ± 0.23	3.29^f^ ± 0.02	5.05^d^ ± 0.03	9.76^a^ ± 0.05
	3	14.10^a^ ± 0.27	10.20^b^ ± 0.28	3.18^f^ ± 0.02	5.65^bc^ ± 0.02	9.89^a^ ± 0.07
18	1	10.35^fg^ ± 0.18	14.83^a^ ± 0.23	3.51^cd^ ± 0.02	5.70^b^ ± 0.24	9.58^b^ ± 0.05
	2	10.58^efg^ ± 0.36	14.83^a^ ± 0.23	3.42^c^ ± 0.03	5.24^d^ ± 0.03	9.68^ab^ ± 0.04
	3	11.85^bc^ ± 0.15	19.13^a^ ± 0.18	3.55^c^ ± 0.01	5.55^cd^ ± 0.08	9.76^a^ ± 0.06
21	1	8.20^h^ ± 0.21	20.10^a^ ± 0.14	3.83^b^ ± 0.04	5.91^b^ ± 0.09	9.18^d^ ± 0.07
	2	8.48^h^ ± 0.42	20.00^a^ ± 0.10	3.75^bc^ ± 0.01	5.13^d^ ± 0.01	9.43^c^ ± 0.06
	3	10.43^fg^ ± 0.27	19.26^a^ ± 0.37	3.65^c^ ± 0.03	5.56^cd^ ± 0.08	9.69^c^ ± 0.05
24	1	7.65^h^ ± 0.12	23.10^a^ ± 0.14	3.97^a^ ± 0.06	5.34^cd^ ± 0.09	9.08^d^ ± 0.06
	2	8.16^h^ ± 0.09	23.13^a^ ± 0.18	3.86^b^ ± 0.01	5.38^cd^ ± 0.02	9.30^cd^ ± 0.07
	3	9.53^g^ ± 0.11	21.93^a^ ± 0.09	3.66^c^ ± 0.03	5.20^d^ ± 0.07	9.61^bc^ ± 0.05

*Note:* Values represent mean ± standard deviation (*n* = 3). Different superscript letters within a column indicate significant differences at *p* < 0.05 (one‐way ANOVA followed by LSD test).

#### 3.3.1 Lactic Acid Production and pH Reduction

Increasing the initial TSS from 9° to 15°Brix resulted in a pronounced enhancement of lactic acid production, indicating improved substrate availability for LAB metabolism. The highest lactic acid concentration (14.10 g/L) was obtained at 15°Brix with a 3% (v/v) inoculum, demonstrating a synergistic effect between elevated fermentable sugar content and higher microbial loading. This outcome reflects enhanced glycolytic flux and accelerated conversion of carbohydrates into lactic acid under optimal substrate–cell density balance. However, further increases in initial TSS beyond 15°Brix (18°–24°Brix) did not lead to proportional increases in lactic acid production. In contrast, lactic acid levels declined at higher TSS values, particularly at 21° and 24°Brix, despite increased residual soluble solids. This phenomenon can be attributed to osmotic stress imposed by high sugar concentrations, which may inhibit LAB growth, reduce membrane integrity, and impair enzymatic activity, ultimately limiting acidification efficiency. Similar inhibitory effects of high osmotic pressure on LAB fermentation kinetics have been reported in fruit and vegetable‐based substrates (Hohmann 2002; Gandhi and Shah 2015; Sionek et al. 2024). The observed trends in lactic acid production were inversely reflected in final pH values. Samples fermented at 15°Brix exhibited the lowest final pH (3.18–3.33), corresponding to the highest acid accumulation, whereas higher °Brix treatments showed elevated pH values (up to 3.97 at 24°Brix), indicating weaker acidification capacity.

#### 3.3.2 Effect of Inoculum Concentration on Microbial Growth

Inoculum concentration significantly affected fermentation dynamics. Increasing the inoculum level from 1% to 3% (v/v) generally accelerated acidification and promoted higher viable LAB counts, particularly at moderate TSS levels (12°–18°Brix). The highest viable cell density (9.89 log CFU/mL) was recorded at 15°Brix with a 3% inoculum, confirming that sufficient initial cell density is essential for rapid adaptation, competitive dominance, and efficient sugar utilization. Nevertheless, excessively high inoculum levels did not consistently improve fermentation outcomes across all TSS conditions. At very high TSS values (21°–24°Brix), increasing the inoculum did not fully overcome the inhibitory effects of osmotic stress, suggesting that substrate composition rather than microbial loading became the limiting factor. These findings align with previous studies indicating that overly concentrated substrates may negate the benefits of higher inoculum levels due to metabolic imbalance and nutrient competition (Wardani et al. 2017; Otite et al. 2024).

#### 3.3.3 Total Phenolic Content

Total phenolic content (TPC) exhibited moderate but significant variation across treatments. Optimal combinations of TSS and inoculum concentration, particularly at 15°Brix and 3% inoculum, resulted in slightly elevated TPC values compared with lower TSS conditions. This increase may be attributed to LAB‐mediated biotransformation of phenolic compounds, including enzymatic hydrolysis of bound phenolics and enhanced extractability during fermentation. Similar increases in phenolic availability during LAB fermentation of vegetable juices have been widely reported (Dissanayake et al. 2025; Kumar et al. 2025). At higher TSS levels, TPC did not increase further and, in some cases, declined slightly, possibly due to oxidative degradation or limited microbial activity under osmotic stress conditions.

#### 3.3.4 Selection of Optimal Conditions

Considering lactic acid production, pH reduction, microbial viability, and phenolic retention collectively, an initial TSS level of 15°Brix combined with a 3% (v/v) inoculum concentration was identified as the optimal condition for carrot juice fermentation. This combination provided a balanced environment that maximized fermentation efficiency without imposing inhibitory stress on LAB cells. Accordingly, these optimized parameters (15°Brix and 3% inoculum) were selected for subsequent fermentation time experiments. While previous sections (Tables 1, 2, 3) report results after a fixed fermentation time of 48 h for comparative and optimization purposes, Section 3.4 further investigates the fermentation kinetics over a broader time range (12–72 h) to better understand temporal changes in biochemical and microbial properties.

### 3.4 Effect of Fermentation Time on Biochemical, Microbial, and Antioxidant Properties

Following the optimization step of fermentation conditions at 48 h, the effect of fermentation time on biochemical, microbial, and antioxidant properties was further evaluated over a range of 12–72 h (Table 4) to elucidate fermentation kinetics. Lactic acid concentration increased steadily from 12 to 72 h, accompanied by a progressive decline in pH, reflecting continuous carbohydrate metabolism and organic acid accumulation by LAB. The most rapid increase in lactic acid production occurred within the first 48 h, reaching 14.03 g/L, after which the rate of increase slowed, indicating a transition toward stationary metabolic activity. Notably, the slight increase in lactic acid concentration observed between 60 and 72 h without a corresponding decrease in pH can be attributed to the buffering capacity of the fermentation system. At low pH values (~3.5), the logarithmic nature of the pH scale limits further detectable changes despite continued acid accumulation. In addition, lactic acid, being a weak organic acid, is only partially dissociated, and the presence of buffering components such as organic acid salts, phenolic compounds, and residual proteins further stabilizes the pH. Similar behavior has been reported in lactic acid fermentation systems, where pH tends to plateau during the later stages of fermentation despite ongoing acid production (Hur et al. 2014). This behavior is consistent with typical LAB fermentation kinetics, where acid accumulation eventually limits further metabolic activity due to self‐inhibition and acid stress (Papadimitriou et al. 2016).

**TABLE 4:** Fermentation characteristics of carrot juice at different fermentation times (12–72 h) under optimized conditions (15°Brix, 3% inoculum).

Fermentation time (h)	12	24	36	48	60	72
Lactic acid (g/L)	8.10 ± 0.18^e^	9.37 ± 0.10^d^	11.70 ± 1.78^c^	14.03 ± 0.21^b^	14.78 ± 0.56^ab^	15.38 ± 0.21^a^
pH	4.07 ± 0.01^a^	3.88 ± 0.01^b^	3.81 ± 0.02^c^	3.66 ± 0.01^d^	3.56 ± 0.01^e^	3.56 ± 0.02^e^
Soluble solids (°Brix)	14.00 ± 0.00^a^	13.17 ± 0.23^b^	13.00 ± 0.00^bc^	12.33 ± 0.23^cd^	11.96 ± 0.12^d^	12.00 ± 0.05^d^
Viable cell count (log CFU/mL)	8.30 ± 0.04^d^	9.00 ± 0.05^c^	9.97 ± 0.06^a^	9.85 ± 0.05^ab^	9.60 ± 0.04^b^	8.95 ± 0.05^c^
Total phenolic content (mg GAE/L)	5.65 ± 0.09^d^	5.77 ± 0.07^cd^	5.86 ± 0.08^c^	6.27 ± 0.10^b^	6.62 ± 0.11^a^	5.78 ± 0.06^cd^
Antioxidant activity (%)	8.63 ± 1.45^d^	9.84 ± 5.01^cd^	11.46 ± 0.89^bc^	12.51 ± 1.51^ab^	14.02 ± 1.43^a^	14.34 ± 2.41^a^

*Note:* Values represent mean ± standard deviation (*n* = 3). Different superscript letters within a row indicate significant differences at *p* < 0.05 (one‐way ANOVA followed by LSD test). Antioxidant activity (%) was measured using the DPPH radical scavenging method.

Viable LAB counts increased rapidly during the early fermentation stage, reaching a maximum at 36 h (9.97 log CFU/mL), followed by a gradual decline at longer fermentation times. The observed decrease in viable cell counts after 48 h can be attributed to increasing acid stress and nutrient depletion. Nevertheless, LAB populations remained above 8.9 log CFU/mL throughout fermentation, exceeding the minimum threshold commonly associated with functional fermented beverages and indicating good microbial stability. Total phenolic content and antioxidant activity exhibited time‐dependent increases, reaching their highest values at 60–72 h. A slight decline in total phenolic content (TPC) was observed alongside an increase in antioxidant activity. Although these parameters are often positively correlated, such divergence is not uncommon in fermented systems and can be attributed to biochemical transformations occurring during fermentation. Enzymatic activities of LAB (e.g., β‐glucosidase and esterase) may convert bound phenolic compounds into smaller molecules with enhanced radical‐scavenging capacity. Furthermore, fermentation can lead to the formation of new bioactive metabolites, including peptides and organic acids, which contribute to antioxidant activity (Hur et al. 2014). It should also be noted that the Folin–Ciocalteu assay measures total reducing capacity rather than specific antioxidant functionality (Singleton et al. 1999), which may not fully reflect the actual changes in antioxidant potential. Consequently, antioxidant activity may increase even when the measured TPC shows a slight decrease. The enhancement of antioxidant capacity is likely associated with LAB‐mediated enzymatic hydrolysis of bound phenolic compounds, as well as the formation of bioactive metabolites during fermentation (Swain et al. 2014; Leonard et al. 2021). Notably, the continued increase in antioxidant activity despite declining viable cell counts suggests that extracellular enzymes and accumulated metabolites remained active during the later fermentation stages. However, prolonged fermentation beyond 48 h resulted in marginal additional gains in antioxidant activity while potentially increasing excessive acidity, which may negatively affect sensory balance. This observation highlights a trade‐off between functional enhancement and sensory quality, where continued acid accumulation at longer fermentation times may compromise consumer acceptability despite biochemical improvements. Such findings emphasize the importance of controlling fermentation duration to achieve an optimal balance between product functionality and palatability. Therefore, considering lactic acid production, microbial viability, antioxidant enhancement, and overall quality attributes, a fermentation duration of 24–48 h was identified as an acceptable range for producing fermented carrot juice with desirable functional properties and sensory acceptability. Among this range, fermentation at 24 h provided the most favorable sensory balance.

### 3.5 Sensory Properties of the Optimized Fermented Carrot Juice

**FIGURE 1:**
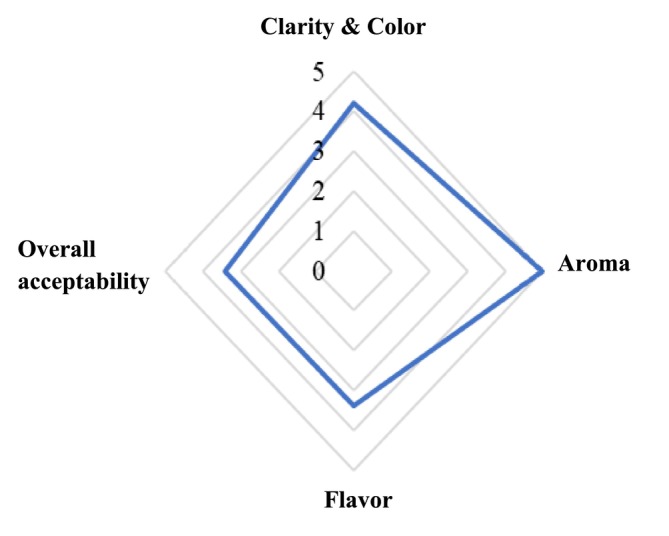
Sensory profile of carrot juice after 24 h of lactic acid fermentation, evaluated using a 5‐point hedonic scale (1 = dislike very much; 5 = like very much; *n* = 9).

The sensory attributes of carrot juice fermented under the optimized conditions (1:1 dilution ratio, 15°Brix, 3% inoculum level, and 24 h fermentation time) were evaluated to assess consumer‐relevant quality characteristics. The sensory profile, illustrated in Figure 1, indicates favorable acceptance across all evaluated attributes, including clarity and color, aroma, flavor, and overall acceptability.

Color received high scores, reflecting the preservation of the natural orange hue of carrot juice and the absence of undesirable turbidity or browning. This result suggests that the selected fermentation conditions effectively minimized pigment degradation and oxidative discoloration, which are common quality issues in vegetable‐based beverages. The relatively high color acceptance is consistent with the moderate fermentation duration (24 h), which limited excessive acidification and pigment instability. Aroma achieved the highest sensory score among all attributes, indicating the formation of pleasant aroma‐active compounds during lactic acid fermentation. LAB‐mediated metabolism is known to generate organic acids and volatile compounds that enhance fresh, mildly acidic, and fruity notes, thereby improving overall aroma perception. The balanced aroma profile observed in this study suggests that fermentation for 24 h promoted desirable flavor compound development without producing off‐odors associated with over‐fermentation. Flavor scores were favorable, indicating good palatability and a well‐balanced sensory profile characterized by a mildly acidic taste, subtle residual sweetness, and a fresh, slightly fruity aroma without detectable off‐flavors. This balance is attributed to moderate lactic acid production after 24 h of fermentation, which contributed to a refreshing taste while avoiding excessive sourness that could negatively affect palatability. The development of desirable volatile compounds during lactic acid fermentation further enhanced aroma perception and overall flavor quality. This finding aligns with the biochemical results, where moderate lactic acid production and stable pH values were observed within the first 24–48 h of fermentation.

Overall acceptability scores confirmed that the optimized fermented carrot juice was well received by the sensory panel, indicating high palatability and consumer acceptance. The harmonious combination of acidity, aroma, and slight sweetness resulted in a pleasant mouthfeel and balanced taste profile. Importantly, sensory evaluation was conducted only at the optimized condition to ensure that the final product met consumer acceptability requirements, rather than during earlier screening or optimization stages. Collectively, these results indicate that lactic acid fermentation can enhance the sensory quality of carrot juice within an optimal fermentation duration. In this study, fermentation at 24 h resulted in a well‐balanced sensory profile, whereas prolonged fermentation beyond this point led to excessive acidification and increased sourness, which may negatively affect flavor balance and palatability. Therefore, the observed balance between functional properties and consumer preference is time‐dependent and limited to the optimized fermentation window, rather than being a general outcome of extended lactic acid fermentation. The favorable sensory profile of the optimized fermented carrot juice supports its potential application as a functional, plant‐based fermented beverage. These results suggest that controlled lactic acid fermentation within the optimized duration can effectively enhance both flavor quality and palatability under optimized conditions of carrot‐based fermented beverages.

## 4 Conclusion

This study systematically evaluated lactic acid fermentation of carrot juice using LAB strains isolated from fermented tomato products and demonstrated that fermentation performance and product quality are strongly influenced by both strain selection and key processing parameters. Among the 10 LAB strains screened, strain TO35 exhibited superior fermentation efficiency, as evidenced by the highest lactic acid production, pronounced pH reduction, and efficient utilization of soluble sugars, and was therefore selected as the optimal starter culture. Juice dilution ratio, initial TSS (°Brix), inoculum concentration, and fermentation time significantly affected the biochemical, microbial, and sensory characteristics of fermented carrot juice. A dilution ratio of 1:1 provided sufficient fermentable substrates while maintaining effective acidification. Increasing the initial TSS to 15°Brix and the inoculum level to 3% (v/v) enhanced lactic acid production, microbial growth, and phenolic retention, whereas excessive sugar concentrations negatively affected fermentation efficiency. Fermentation time strongly influenced metabolic dynamics, with lactic acid accumulation, pH reduction, and antioxidant activity increasing over time, while viable LAB counts peaked at intermediate stages and declined slightly thereafter. Based on an integrated assessment of fermentation performance and sensory acceptability, a fermentation duration of 24 h was identified as the most suitable condition in terms of sensory quality within the acceptable fermentation range. This study has several limitations. The LAB strains were identified based on phenotypic and biochemical characteristics without molecular confirmation at the species level. In addition, detailed metabolite profiling was not performed. Future studies should incorporate molecular identification and advanced analytical approaches to provide deeper insight into strain‐specific functionality and fermentation mechanisms. Sensory evaluation confirmed that the optimized fermented carrot juice exhibited a balanced and favorable sensory profile, with high scores for color, aroma, flavor, and overall acceptability. Overall, this study provides a useful framework for optimizing lactic acid fermentation of carrot juice and supports its potential application as a clean‐label, functional, plant‐based fermented beverage. These findings offer valuable insights for both academic research and industrial development of nutritious, microbiologically stable, and sensorially acceptable fermented vegetable products.

## Author Contributions

**Thanh Viet Nguyen:** conceptualization, methodology, validation, formal analysis, investigation, data curation, writing – original draft, visualization, writing – review and editing. **Chi Khang Van:** methodology, validation, formal analysis, visualization. **Ho Thi Uyen Em:** methodology, validation, formal analysis, visualization. **Luu Minh Chau:** methodology, validation, formal analysis, visualization. **Nguyen Ngoc Thanh:** methodology, validation, formal analysis, visualization. **Huynh Xuan Phong:** conceptualization, methodology, validation, formal analysis, investigation, data curation, visualization, supervision, project administration, funding acquisition, writing – review and editing.

## Ethics Statement

Ethical approval was not required for this study according to the institutional policy of Can Tho University. All participants involved in the sensory evaluation provided informed consent prior to participation.

## Conflicts of Interest

The authors declare no conflicts of interest.

## Data Availability

The authors confirm that data supporting the findings of this study are available within the article. Raw data are available from the corresponding author upon reasonable request.
